# Neuropathy caused by pesticide exposure on farmers in Ngablak District, Magelang, Central Java, Indonesia: An electroneuromyography study

**DOI:** 10.1016/j.toxrep.2023.09.020

**Published:** 2023-10-03

**Authors:** Andre Stefanus Panggabean, Ismail Setyopranoto, Arjanto Ramadian Wicaksono, Alfian Rismawan, Ery Kus Dwianingsih, Whisnu Nalendra Tama, Abdul Gofir, Cempaka Thursina Srie Setyaningrum, Sri Sutarni, Ahmad Asmedi, Aulia Fitri Rhamadianti, Rheza Gandi Bawono, Rusdy Ghazali Malueka

**Affiliations:** a Neurology Department, Faculty of Medicine, Public Health, and Nursing, Universitas Gadjah Mada, Dr. Sardjito General Hospital, Yogyakarta, Indonesia; b Department of Anatomical Pathology, Faculty of Medicine, Public Health and Nursing, Universitas Gadjah Mada, Dr. Sardjito General Hospital, Yogyakarta, Indonesia

**Keywords:** Pesticides, NCS, Organophosphate, Carbamate, Neurophysiology

## Abstract

Uncontrolled and unsafe use of pesticides can lead to acute and chronic toxicity in farmers, with neuropathy being one of the most common symptoms of chronic toxicity. However, the effects of this toxicity on farmers' electroneuromyography (ENMG) are still unclear. To address this, we conducted a cross-sectional study from July to October 2017 in Ngablak District, Magelang, Central Java, Indonesia. Eligible farmers who were exposed to pesticides underwent electrophysiology examinations, as well as additional tests such as physical examination and laboratory testing. We collected general information such as age and work history by interview. In total, 64 farmers were included in this study. Out of these, 44 farmers were found to have polyneuropathy, with 41 of them having motor polyneuropathy and 19 of them having sensory polyneuropathy. Our findings showed that low blood cholinesterase was associated with distal latency prolongation (p-value: 0.014). The group exposed to organophosphate/carbamate pesticides was also significantly associated with prolonged distal latency (p-value: 0.012). However, motor polyneuropathy was significantly associated with chronic exposure to organophosphate/carbamate pesticides (p-value: 0.009) and not with low blood cholinesterase levels (p-value: 0.454). The study concludes that chronic exposure to organophosphate or carbamate pesticides could result in polyneuropathy disease, particularly in the motor system.

## Highlights

• Most of the farmers exposed to pesticides had polyneuropathy in this study.
• Chronic exposure to organophosphate/carbamate was associated with polyneuropathy.
• Low blood cholinesterase levels were not associated with polyneuropathy.

## 1 Introduction

Pesticides have been used widely in agricultural fields to increase crop and food production, especially in major agricultural nations such as Indonesia. Farmers are routinely using pesticides to control pests and diseases of the crops. In the last three decades, the widespread use of pesticides has rapidly increased [1]. This overuse could lead to several serious medical problems. Chronic exposure to organophosphates, one category of pesticides, has been linked to many debilitating diseases, including diabetes mellitus [2], Alzheimer’s disease [3], breast cancer, and thyroid cancer [4]. In neurological systems, chronic exposure could induce neuropsychological impairment in cognitive function, memory, attention, coordination, and many more functional aspects [5]. Carbamate, another cholinesterase inhibitor pesticides, could cause psychomotor and neuropsychiatric problems in its long-term effects [6].

Pesticide poisoning could be influenced by internal and external risk factors. Internal risk factors include age, sex, nutritional status, hemoglobin status, health condition, and education. On the other hand, external aspects of pesticide poisoning include pesticide dose, environmental temperature, wind direction, concentration and potency of pesticides, duration of pesticide exposure, duration of spraying, frequency of spraying, protective equipment use, and type of pesticides [7], [8].

Organophosphate Induced Delayed Neuropathy (OPIDN) is a disease caused by organophosphate pesticides poisoning. OPIDN is characterized by a progressive distal axonopathy of peripheral nerves and spinal cord, usually induced by acute high-dose intoxication [9]. A chronic low-dose exposure (such as occupational exposure) effect on peripheral nerve or causing OPIDN is relatively new, and the evidence is limited.

Some articles have studied the pesticide effect on peripheral nerves using electrophysiology study. The results were mixed; Some showed prolonged distal latency, reduced motor and sensory nerve conduction velocity, and reduced motoric amplitude in the exposed group. Other studies showed low-dose organophosphate pesticides exposure is not always associated with peripheral neurophysiology impairment [10]. Based on these findings, this study was conducted to assess the electrophysiologic parameters of farmers chronically exposed to pesticides in Ngablak District, Magelang, Indonesia, and compared other risk factors that could influence those abnormalities.

## 2 Materials and methods

### 2.1 Study sample

This cross-sectional analytic study was conducted from July to October 2017 in Ngablak District, Magelang, Central Java, Indonesia, and Sardjito Hospital, Yogyakarta. Seloprojo village was selected based on the principle of stratified random sampling. In total, 64 farmers were chosen based on inclusion and exclusion criteria. The inclusion criteria were: 1) Farmers who used pesticides in the last two weeks and have been using them for at least two weeks; and 2) willing to be brought to Dr. Sardjito Hospital for an electrophysiology examination. Farmers with other diseases that could induce peripheral neuropathy were excluded, such as diabetes mellitus, chronic alcohol use, amputation of extremities, and foot ulcer. All included farmers were informed about the study procedure and asked to sign the informed consent forms after they understood the process. This study was approved by the Institutional Ethics Committee of the Faculty of Medicine, Public Health and Nursing, Universitas Gadjah Mada, with ethical clearance number KE/FK/0340/EC/2017).

### 2.2 Subject characteristics, pesticide exposure, personal protective equipment, and knowledge

Subjects’ characteristics were assessed from interviews (age, length of work as a farmer) and physical examination (Body Mass Index). The research team developed a questionnaire to determine the exposure to pesticides. The questionnaire covers various aspects of pesticide usage, such as time, duration, and frequency of spraying, the brand and type of pesticides used, methods for pesticide preparation, and more. The type of pesticide used by farmers was determined from the reported brand, and the active ingredients were identified through the pesticide label, Material Safety Data Sheet (MSDS), and data from the National Institute for Occupational Safety and Health Institute (NIOSH). Personal Protective Equipment (PPE) scores were assessed by asking if they used each of the 8 PPE equipment the World Health Organization recommended [11]. For every component used, subjects got 5 points, and the scoring range was 0 – 40. Pesticide knowledge was determined using a questionnaire developed by the research team, as shown in the Appendices.

### 2.3 Blood cholinesterase levels

Blood samples were collected and stored in EDTA vacutainer tubes. The tubes were transported immediately to a private laboratory using wet ice to be analyzed. Ellman’s method was used to determine cholinesterase levels [12]. Normal blood cholinesterase levels range from 9.572 to 15.031 IU/mL. A level below 9.572 is considered a Low Blood Cholinesterase Level (LBCL) [13].

### 2.4 Nerve conduction study (NCS)

All subjects were brought to Dr. Sardjito Hospital in Yogyakarta. This study used Nihon Kohden Neuropack X1 EMG/EP Measuring System MEB-2300K for NCS examination. All four extremities were studied. In the upper extremities, Compound Muscle Action Potential (CMAP) and Sensory Nerve Action Potential (SNAP) in median nerves and ulnar nerves were recorded; for lower extremities, CMAP was recorded from tibial nerves and SNAP was recorded from sural nerves.

The results were shown in numeric data and compared to other variables using categorical data. We divided the NCS results into two groups, normal and abnormal, based on Liveson’s normal results [14]. Distal latency (DL) abnormality was diagnosed if the results were higher than the upper limit of normal values. Nerve conduction velocity (NCV)abnormality was diagnosed if the results were lower than the lower limit of the normal value. For distal amplitude (DA) and proximal amplitude (PA), the abnormality was diagnosed if the results were lower than the lower limit of the normal value. To conclude abnormal results in motor DL, the subjects must have abnormal distal latency results in all three nerves (median, ulnar, and tibial nerve). This algorithm is also used for nerve conduction velocity (NCV), distal amplitude (DA), and proximal amplitude (PA). This algorithm was used to eliminate the possibility of subclinical mononeuropathy caused by ergonomic problems in farmers’ daily activity, such as carpal tunnel syndrome [15]. For sensory DL and DA, there must be abnormal results in all three nerves (median, ulnar, and sural). Motor polyneuropathy was diagnosed if the subject had abnormal results in all four motor nerve markers: DL, NCV, DA, and PA. In contrast, sensory polyneuropathy was diagnosed if the subjects had abnormal sensory DL and DA results. If the subjects had either motor or sensory abnormal results, the subject was said to have polyneuropathy. We categorize the results into demyelination or axonal degeneration to understand the possible mechanism. Demyelination was diagnosed if there was slower NCV (< 75% than the lower limit of normal scores), prolonged DL (> 130% than the upper limit of normal scores), or both. In contrast, axonal degeneration was diagnosed if CMAP or SNAP amplitude was lower than the lower limit of normal scores.

### 2.5 Statistical analysis

Descriptive data was used to present results, which were then categorized into Normal and Low Blood Cholinesterase Levels (LBCL) groups based on cholinesterase examination. The two pesticide exposure groups were distinguished based on the type of pesticide exposure: chronic exposure to organophosphate/carbamate pesticides or exposure to other pesticide types**.** Shapiro-Wilk testing was used to determine the normality of data distribution. Data with normal distribution were shown using mean ± standard deviation (SD), whereas the median (minimum–maximum) was used for abnormal distribution. Next, we used an independent t-test (parametric) or Mann-Whitney (non-parametric) to test categorical-numerical association and a chi-square test to test categorical-categorical association. Last, to exclude age, sex, body mass index (BMI), length of work, and farmers' knowledge, we used Binomial Logistic Regression to find any association with the NCS results.

## 3 Results

There were 64 subjects included in this study based on inclusion and exclusion criteria. The sample population was 81% males and 19% females. The subjects’ characteristics are shown in Table 1. The mean age ± SD in this study was 47.94 ± 10.96. The median length of work was 21.5 years. In the PPE score, the median was 20, with 5 as the lowest and 35 as the highest score. This meant nobody either used all the PPE or did not use any of the PPE. The knowledge median score was 17.5, ranging between 6 and 45. The BMI mean was 22.73 with 3.37 SD, and the blood cholinesterase level mean was 8.49 with 1.50 SD. In this study, 42.2% of farmers used organophosphate or carbamate pesticides types, and 57.8% did not use these two types.

**Table 1:** Subjects’ characteristics (n = 64).

Variable	n (%)	Means (SD)	Median (Min-Max)	95% CI
**Age**	-	47.94 + 10.96	-	45.20–50.68
**Sex**				
• **Male**	52 (81.25)	-	-	-
• **Female**	12 (18.75)	-	-	-
**Length of work (years)**	-	-	21.5 (2–70)	21.61–29.86
**PPE Scores**	-	-	20 (5–35)	17.87–21.66
**Pesticide Knowledge Scores**	-	-	17.5 (6–45)	17.73–22.62
**Body Mass Index (BMI)**	-	22.73 + 3.37	-	21.88–23.56
**Pesticide Types**				
• **Organophosphate / Carbamate**	27 (42.2)	-	-	-
• **Other Types**	37 (57.8)	-	-	-
**Cholinesterase Levels**	-	8.49 + 1.50	-	8.11–8.86

CI, confidence interval; PPE, personal protective equipment; SD, standard deviation.

Table 2 displays the mean or median of each examination. In the case of the median nerve, the median of CMAP and SNAP DL were longer than the normal range, but their NCV median, CMAP DA, and CMAP PA mean were normal. The ulnar nerve's SNAP DL was below the normal range. However, the tibial and sural nerve's mean or median were within the normal range.

**Table 2:** Nerve conduction study data (n = 64).

Variable	Means (SD)	Median (Min-Max)	95% CI
**CMAP Median Nerve**			
**DL**	-	4.31 (2.92–12.9)	4.3–5.11
**NCV**	-	53.15 (26.3–65.4)	50.01–53.74
**DA**	8.53 + 2.73	-	7.85–9.21
**PA**	8.39 + 2.91	-	7.66–9.12
**CMAP Ulnar Nerve**			
**DL**	-	2.81 (1.80–14.71)	2.82–3.67
**NCV**	-	59.7 (46.3–78.5)	58.1–61.53
**DA**	7.21 + 2.53	-	6.58–7.84
**PA**	7.08 + 2.01	-	6.57–7.58
**CMAP Tibial Nerve**			
**DL**	-	4.67 (3.20–12.60)	4.61–5.32
**NCV**	40.63 + 4.65	-	39.47–41.79
**DA**	9.01 + 4.05	-	8.01–10.02
**PA**	-	6.64 (1.25–15.2)	6.61–8.34
**SNAP Median Nerve**			
**DL**	-	3.4 (2.36–6.33)	3.34–3.76
**DA**	-	8.5 (0–36)	8.62–12.89
**SNAP Ulnar Nerve**			
**DL**	-	2.6 (1.78–6.0)	2.55–2.82
**DA**	-	10.5 (0–49)	9.30–13.77
**SNAP Sural Nerve**			
**DL**	-	2.37 (1.4–4.4)	2.38–2.75
**DA**	-	10.50 (0–61)	10.33–15.83

Normal Range: **Distal Latency (DL)** for motor: Median Nerve: 3.15–3.83 ms; Ulnar Nerve: 1.9–2.98 ms; Tibial Nerve: 2.96–4.96 ms. **Nerve Conduction Velocity (NCV):** Median Nerve: 49.7–69.1 m/s; Ulnar nerve: 53.6–73.0 m/s; Tibial nerve: 44.1–52.1 m/s; **Distal Amplitude (DA)** for motor**:** Median Nerve: > 4 mV; Ulnar Nerve: > 3.7 mV; Tibial Nerve: > 3.9 mV; **Proximal Amplitude (PA)** for motor**:** Median Nerve: > 4 mV; Ulnar Nerve: > 3.5 mV; Tibial Nerve: > 2.9 mV; **Distal Latency (DL)** for sensory**:** Median Nerve: < 3.18 ms; Ulnar Nerve: < 2.83 ms; Sural Nerve: 2.8–4.4 ms; **Distal Amplitude (DA)** for sensory**:** Median Nerve: > 22.9 uV; Ulnar Nerve: > 18 uV; Sural Nerve: > 5.0 uV. CMAP, Compound Muscle Action Potential; SNAP, Sensory Nerve Action Potential.

From blood cholinesterase levels examination, we found 45 subjects (70.3%) in the LBCL group and only 19 subjects (29.7%) in normal cholinesterase groups. When compared with the normal cholinesterase groups, we found slower NCV of tibial nerves, longer motor DL for median nerves and tibial nerves, longer sensory DL of ulnar nerves and sural nerves, and lower motor amplitude of median nerves, ulnar nerves, and tibial nerves in LBCL groups. Still, these differences were not statistically significant (Table 3).

**Table 3:** Nerve conduction study differences between LBCL and normal cholinesterase groups (n = 64).

	Normal Cholinesterase Group	LBCL Group	*p-*value
**CMAP Median Nerve**			
**DL**	4.61 ± 1.49	4.74 ± 1.67	0.566
**NCV**	51.61 ± 8.25	51.99 ± 7.22	0.763
**Amplitude**	9.03 ± 2.28	8.32 ± 2.89	0.243
**CMAP Ulnar Nerve**			
**DL**	3.70 ± 2.87	3.05 ± 0.80	0.612
**NCV**	59.68 ± 6.22	59.90 ± 7.10	0.889
**Amplitude**	7.92 ± 2.86	6.91 ± 2.34	0.378
**CMAP Tibial Nerve**			
**DL**	4.68 ± 1.02	5.08 ± 1.54	0.303
**NCV**	40.72 ± 4.48	40.59 ± 4.77	0.843
**Amplitude**	9.09 ± 3.70	8.98 ± 4.22	0.681
**SNAP Median Nerve**			
**DL**	3.56 ± 0.90	3.55 ± 0.83	0.843
**Amplitude**	8.13 ± 6.54	11.86 ± 9.09	0.147
**SNAP Ulnar Nerve**			
**DL**	2.57 ± 0.34	2.73 ± 0.61	0.474
**Amplitude**	10.44 ± 7.39	12.00 ± 9.56	0.663
**SNAP Sural Nerve**			
**DL**	2.43 ± 0.43	2.62 ± 0.82	0.763
**Amplitude**	11.71 ± 8.77	13.66 ± 11.89	0.653

Normal Range: **Distal Latency (DL)** for motor: Median Nerve: 3.15–3.83 ms; Ulnar Nerve: 1.9–2.98 ms; Tibial Nerve: 2.96–4.96 ms. **Nerve Conduction Velocity (NCV):** Median Nerve: 49.7–69.1 m/s; Ulnar nerve: 53.6–73.0 m/s; Tibial nerve: 44.1–52.1 m/s; **Distal Amplitude (DA)** for motor**:** Median Nerve: > 4 mV; Ulnar Nerve: > 3.7 mV; Tibial Nerve: > 3.9 mV; **Proximal Amplitude (PA)** for motor**:** Median Nerve: > 4 mV; Ulnar Nerve: > 3.5 mV; Tibial Nerve: > 2.9 mV; **Distal Latency (DL)** for sensory**:** Median Nerve: < 3.18 ms; Ulnar Nerve: < 2.83 ms; Sural Nerve: 2.8–4.4 ms; **Distal Amplitude (DA)** for sensory**:** Median Nerve: > 22.9 uV; Ulnar Nerve: > 18 uV; Sural Nerve: > 5.0 uV. CMAP, Compound Muscle Action Potential; LBCL, Low Blood Cholinesterase Levels; SNAP, Sensory Nerve Action Potential.

When the subjects were divided into the organophosphate/carbamate pesticides exposed group and other pesticides group, subjects who were exposed to organophosphate or carbamate pesticides had prolonged median and ulnar CMAP DL, and lower median and tibial CMAP amplitude (Table 4). However, these differences were not statistically significant (*p* > 0.05).

**Table 4:** Nerve conduction study differences between organophosphate/carbamate pesticides and other pesticides (n = 64).

	Organophosphate/Carbamate Pesticides	Others Pesticides	*p-*value
**CMAP Median Nerve**			
**DL**	5.01 ± 1.97	4.48 ± 1.26	0.180
**NCV**	52.17 ± 7.77	51.67 ± 7.36	0.536
**Amplitude**	8.03 ± 2.65	8.89 ± 2.76	0.100
**CMAP Ulnar Nerve**			
**DL**	3.49 ± 2.39	3.07 ± 0.93	0.501
**NCV**	61.84 ± 7.44	58.37 ± 5.98	0.109
**Amplitude**	7.41 ± 2.30	7.07 ± 2.70	0.301
**CMAP Tibial Nerve**			
**DL**	4.86 ± 1.01	5.04 ± 1.65	0.935
**NCV**	40.06 ± 4.16	41.05 ± 5.00	0.422
**Amplitude**	8.53 ± 4.08	9.36 ± 4.04	0.467
**SNAP Median Nerve**			
**DL**	3.52 ± 0.89	3.57 ± 0.82	0.549
**Amplitude**	11.49 ± 8.25	10.22 ± 8.82	0.438
**SNAP Ulnar Nerve**			
**DL**	2.63 ± 0.34	2.72 ± 0.75	0.643
**Amplitude**	14.85 ± 11.16	9.12 ± 5.98	0.034
**SNAP Sural Nerve**			
**DL**	2.49 ± 0.71	2.62 ± 0.75	0.466
**Amplitude**	12.85 ± 11.75	13.25 ± 10.62	0.759

Normal Range: **Distal Latency (DL)** for motor: Median Nerve: 3.15–3.83 ms; Ulnar Nerve: 1.9–2.98 ms; Tibial Nerve: 2.96–4.96 ms. **Nerve Conduction Velocity (NCV):** Median Nerve: 49.7–69.1 m/s; Ulnar nerve: 53.6–73.0 m/s; Tibial nerve: 44.1–52.1 m/s; **Distal Amplitude (DA)** for motor**:** Median Nerve: > 4 mV; Ulnar Nerve: > 3.7 mV; Tibial Nerve: > 3.9 mV; **Proximal Amplitude (PA)** for motor**:** Median Nerve: > 4 mV; Ulnar Nerve: > 3.5 mV; Tibial Nerve: > 2.9 mV; **Distal Latency (DL)** for sensory**:** Median Nerve: < 3.18 ms; Ulnar Nerve: < 2.83 ms; Sural Nerve: 2.8–4.4 ms; **Distal Amplitude (DA)** for sensory**:** Median Nerve: > 22.9 uV; Ulnar Nerve: > 18 uV; Sural Nerve: > 5.0 uV. CMAP, Compound Muscle Action Potential; LBCL, Low Blood Cholinesterase Levels; SNAP, Sensory Nerve Action Potential.

From the bivariable analysis (Table 5), there were no significant differences between LCBL and normal cholinesterase groups in age, sex, BMI, length of work, PPE Scores, and Knowledge Scores (*p* > 0.05). These same results were found for the organophosphate/carbamate pesticides and other pesticide groups (*p* > 0.05) (Table 6).

**Table 5:** Bivariable analysis comparing LCBL and normal cholinesterase groups based on cholinesterase levels (n = 64).

Variable	LBCL Groups	Normal Cholinesterase Groups	*p*	Mean Differences/OR (95% CI)
Age (Mean)^a^	50.16	46.31	0.249	3.84 (- 2.78 – 10.46)
Sex^b^				
• Male	37	15	0.759	1.233 (0.322 – 4.719)
• Female	8	4		
Length of Work (Mean)^c^	28.20	19.89	0.105	8.30 (- 0.54 – 17.15)
Body Mass Index (Mean)^a^	22.24	23.85	0.112	- 1.611 (- 3.61 – 0.39)
PPE Scores (Mean)^c^	20.11	18.94	0.211	1.16 (- 3.11 – 5.44)
Pesticide Knowledge Scores (Mean)^c^	20.87	18.52	0.590	2.34 (- 3.47 – 8.15)

CI, confidence interval; LCBL, Low Blood Cholinesterase Levels; OR, odds ratio; PPE, personal protective equipment; *p-value < 0.05 considered significant.*

a Using Independent t-test analysis;

b Chi-square test analysis;

c Mann-Whitney test analysis.

**Table 6:** Bivariable analysis comparing organophosphate/carbamate pesticides exposed group with other pesticides type group (n = 64).

Variable	Organophosphate/Carbamate Pesticides	Other Types	*p*	Mean Differences/OR (95% CI)
Age (Mean)	49.22	48.86	0.910	0.35 (-5.90–6.62)
Sex				
• Male	20	32	0.209	0.446 (0.125–1.600)
• Female	7	5		
Length of Work (Mean)	23.59	27.29	0.633	- 3.70 (-11.63–4.22)
Body Mass Index (Mean)	22.14	23.14	0.229	- 0.99 (-2.63–0.64)
PPE Scores (Mean)	20.37	19.32	0.568	1.04 (-2.81–4.90)
Pesticide Knowledge Scores (Mean)	20.11	20.21	0.400	- 0.10 (-4.82–4.61)

^a^ Using Independent t-test analysis; ^b^Chi-square test analysis; ^c^Mann-Whitney test analysis;

CI, confidence interval; OR, odds ratio; PPE, personal protective equipment; *p*-value < 0.05 was considered significant.

We could determine the amount of abnormality in the subjects (Table 7). There were 44 subjects with polyneuropathy, of which 41 of them had motor abnormalities and 19 of them had sensory abnormalities. Based on the indices, we found the CMAP DL was the highest, with 36 farmers had abnormality in motor DL. There were only 5 farmers with NCV abnormality, 5 farmers with SNAP DL abnormality, and 9 farmers with SNAP amplitude abnormality.

**Table 7:** Abnormality of nerve conduction study in all groups (n = 64).

	(n) abnormal	%
**Overall Polyneuropathy**	44	68.8
**Motor polyneuropathy**	41	64.1
**Sensory polyneuropathy**	19	29.7
**Motor**		
– **Distal Latency**	36	56.3
– **Nerve Conduction Velocity**	5	7.8
– **Distal Amplitude**	0	0
– **Proximal Amplitude**	0	0
**Sensory**		
– **Distal Latency**	5	7.8
– **Distal Amplitude**	9	14.1
**Nerves**		
– **Median Nerve**	50	78.1
– **Ulnar Nerve**	44	68.8
– **Tibial Nerve**	54	84.4
– **Sural Nerve**	24	37.5
**Demyelination**		
– **Overall**	7	10.9
– **Motor**	7	10.9
– **Sensory**	5	7.8
**Axonopathy**	9	14.1

Next, we divided the NCS results into normal and abnormal results and we compared them based on their cholinesterase levels and pesticide types (Table 8). Motor DL was the only one parameter that had significant association with LBLC groups (p = 0.014) with odds ratio (OR) of 5.046. There was no significant association between the other parameters with low blood cholinesterase levels. In this analysis, we did not analyze CMAP amplitude because of zero amplitude abnormalities.

**Table 8:** The association between low blood cholinesterase levels with abnormality in ENMG markers (n = 64).

NCS	Low Blood Cholinesterase Levels		
	Adjusted odds ratio	Adjusted *p*-value	95% CI
Polyneuropathy			
Overall	1.627	0.454	0.455–5.825
Motor	2.284	0.186	0.671–7.774
Sensory	0.838	0.801	0.213–3.299
Markers			
Motor			
– DL	5.046	0.014*	1.379–18.470
– NCV	1.725	0.645	0.170–17.530
Sensory			
– DL	1.392	0.785	0.129–15.01
– DA	0.248	0.106	0.046–1.342
DL overall	3.885	0.036*	1.092–13.816
Nerve			
– Median	2.457	0.172	0.677–8.919
– Ulnar	5.795	0.044*	1.049–32.002
– Tibial	0.445	0.356	0.080–2.483
– Sural	0.963	0.955	0.254–3.641
Demyelination			
– Motor	1.969	0.522	0.248–15.662
– Sensory	0.352	0.183	0.076–1.634
Overall	1.969	0.522	0.248–15.662
Axonopathy	0.248	0.106	0.046–1.342

All variables were adjusted with age, sex, BMI, length of work, PPE scores, and knowledge scores. CI, confidence interval; LBCL, Low Blood Cholinesterase Levels.

* *p*-value < 0.05 was considered significant.

Next, we found a significant association between organophosphate or carbamate pesticides chronic use with NCS abnormality (Table 9). Motor distal latency abnormality was found higher in the exposed group (*p* = 0.012) with OR of 4.598. No significant association was found with NCV, sensory DL, or DA. Combining all of the NCS results, we found a 440% (ORs: 5.406, *p*-value=0.009) increase in OR for motor polyneuropathy and 640% (ORs: 7.408; p-value=0.009) increase in OR for overall polyneuropathy in organophosphate/carbamate pesticides chronic exposed groups.

**Table 9:** The Association between organophosphate/carbamate pesticides chronic exposed group with abnormality in ENMG markers (n = 64).

NCS	Organophosphate/Carbamate Pesticides Chronic Exposed Group		
	Adjusted odds ratio	Adjusted *p*-value	95% CI
Polyneuropathy			
Overall	7.408	0.009*	1.642–33.411
Motor	5.406	0.009*	1.521–19.216
Sensory	0.753	0.656	0.216–2.624
Markers			
Motor			
– DL	4.598	0.012*	1.403–15.071
– NCV	3.003	0.272	0.434–25.659
Sensory			
– DL	0.408	0.448	0.040–4.127
– DA	0.150	0.090	0.017–1.344
DL overall	4.837	0.013*	1.389–16.841
Nerves			
– Median	4.583	0.043*	1.051–19.99
– Ulnar	0.316	0.143	0.068–1.476
– Tibial	11.22	0.034*	1.195–105.4
– Sural	0.930	0.901	0.295–2.924
Demyelination			
– Motor	1.052	0.814	0.206–7.467
– Sensory	0.408	0.448	0.040–4.127
Overall	1.052	0.814	0.206–7.467
Axonopathy	0.150	0.090	0.017–1.344

All variables were adjusted with age, sex, BMI, length of work, PPE scores, and knowledge scores. CI, confidence interval.

* *p*-value < 0.05 was considered significant.

The LBCL groups exposed to organophosphate or carbamate pesticides had a higher OR to have demyelination injury, but this was not significant (*p* = 0.522 and *p* = 0.814, respectively). There were also no significant associations with axonopathy mechanism (*p* = 0.106 and *p* = 0.090, respectively).

## 4 Discussion

Blood cholinesterase levels had a normal distribution with mean of 8.49 ± 1.5. These results were below the normal range (9.572 U/L) [13]. There were 70.3% of subjects who had low cholinesterase levels, which is similar to a prior study [7] with 7.02% who had been poisoned. Organophosphate and organochlorine type of pesticides inhibit pseudocholinesterase in plasma cholinesterase in red blood cells and in the synapse, which at a certain level cause poisoning. Carbamate also inhibits acetylcholinesterase but only causes reversible inhibition of butyrylcholinesterase [16]. Other pesticides, such as pyrethroid (which works in sodium and chloride channels) do not influence the cholinesterase levels [17]. This study did not find any significant association between blood cholinesterase and NCS results.

Some studies showed similar results. In one study, there was no significant association found between decreasing blood cholinesterase before and after spraying with NCS [18]. Differing from organophosphate pesticides acute intoxication, the pathophysiology of polyneuropathy was not related to the inhibition of cholinesterase levels but was instead caused by the inhibition of neuropathy target esterase (NTE) [19]. As a result, this could explain why the severity of neuropathy was not correlated with the low cholinesterase levels.

In this study, we found a significant association between chronic exposure to organophosphate or carbamate pesticides with polyneuropathy, especially in the motor system, but not in the sensory system. Results on NCS were relatively mixed. One study did not find any difference in NCS results between baseline and after 1 year [9]. A study in Iran on chronic low-dose pesticide exposure showed a slower peroneal NCV, longer sural and radial SNAP latency, and smaller sural and radial SNAP amplitude [20]. The reason for these inconsistent results is due to the different study types, duration, and interpretation of NCS results. Farming and spraying are occupations that require immense energy expenditure each day and when a study showed abnormality in one nerve but not in the other, there was a possibility that subclinical mononeuropathy happened because of high work force [15]. Another problem caused by this was the difficulty obtaining a bigger picture and explanation of what happened if the lesion happened only in one nerve but not in another (i.e. why the NCV in peroneal is significantly slower but not in sural). Our study tried to conclude the result of each subject into a diagnosis (polyneuropathy), so we can be sure that pesticide was the cause, not muscle overuse. One prospective study [21] also tried to conclude all the results into a whole picture and that study showed there was significantly higher ratio of NCV abnormality but not in distal latency or amplitude. The study used the OR formula (an NCS index was abnormal if one or more parameters were out of normal range) while our study used the AND formula for each NCS marker (an NCS index was abnormal if all nerves in that index had abnormal results). Again, with the AND formula, we could exclude subclinical mononeuropathy lesions that are usually caused by nerve entrapment.

The neurotoxic effects of organophosphate or carbamate pesticides may be explained by their photodegradation properties. Organophosphate pesticides degrade relatively quickly in the environment [22]. However, their degradation metabolites are more toxic than the original compound. 3,5,6-trichloropyridinol (TCP) is one of the main metabolites of chlorpyrifos, one of the most commonly used organophosphate pesticides. TCP is even more toxic than chlorpyrifos itself [23]. This metabolite can permeate into the soil and water, leading to indirect exposure of farmers.

The mechanism of neuropathy by organophosphate and carbamate pesticides usually begins with axonal degeneration followed by secondary demyelination [24]. In this study, we could only find a higher OR to have demyelination injury in LBCL and organophosphate/carbamate pesticides exposed group, but this result was not significant. This finding could lead to another possible mechanism that induced demyelination without abnormal amplitude results. A prospective study [21] showed organophosphate pesticides did not produce a higher risk in SNAP and CMAP amplitude abnormality but it did produce abnormality in NCV.

This study has several limitations that should be considered when interpreting the results. Firstly, the study did not categorize the participants according to their exposure to pesticides, i.e. high dose versus low dose. This lack of categorization is important, as it is possible that a high dose of pesticide may lead to a different mechanism of toxicity, such as OPIDN, compared to a low dose. Secondly, there may have been some memory bias among the participants, as many of them had worked in farming for an average of 21.5 years. As a result, they may have only remembered the last pesticide that they used, and may have forgotten about previous exposure to organophosphate or carbamate pesticides. To mitigate this bias, the researchers identified all organophosphate and carbamate pesticides products in the area and asked the participants if they had used any of those products. If the participants answered "no", their response was categorized as "other pesticide type". Future studies should consider examining metabolites of organophosphate pesticides such as diphenyl phosphate and bis(1,3-dichloro-2-propyl) phosphate [25]. Finally, environmental conditions such as temperature changes, precipitation, sunlight radiation, and air movement could affect the degradation of pesticides, which in turn could influence the toxicity mechanism of the pesticide [26]. Hence, these factors should be considered in future studies.

## 5 Conclusion

This study found that chronic exposure to organophosphate or carbamate pesticides could cause polyneuropathy, mainly in the motor system. However, this neuropathy was not associated with low blood cholinesterase levels.

## Funding

This study was supported by the Ministry of Research and Technology/National Research and Innovation Agency of the Republic of Indonesia under University Excellence in Research Grant.

## Ethical consideration

This study was reviewed and approved by the Medical and Health Research Ethics Committee of the Faculty of Medicine, Public Health and Nursing, Universitas Gadjah Mada with approval number of KE/FK/0340/EC/2017.

## CRediT authorship contribution statement

**Andre Stefanus Panggabean:** Coordinating Research Conduction in Ngablak and in Neurophysiology Department. **Ismail Setyopranoto:** Conceptualization, Investigation in Ngablak, Writing – original draft. **Arjanto Ramadian Wicaksono**: Investigator of Demographic Data and Conducting ENMG examination. **Alfian Rismawan**: Data Manager, Analysis, and Writing – original draft. **Ery Kus Dwianingsih**: Conceptualization, Methodology, Investigator of Demographic Data in Ngablak. **Whisnu Nalendra Tama**: Methodology, Investigator of Demographic Data and Examination in Ngablak. **Abdul Gofir**: Investigator, Data Administrator, and Investigator of Physical Examination in Ngablak. **Cempaka Thursina Srie Setyaningrum**: Investigator of Demographic Data and Examination in Ngablak, Data Administration, Review & Editing of Manuscript **Sri Sutarni**, **Ahmad Asmedi**: Investigator of ENMG in Dr. Sardjito Hospital, Conceptualization, Supervision, Resources, Methodology, Review & Editing of Manuscript. **Aulia Fitri Rhamadianti**: Data Administration, Data Analysis, Writing – review & editing. **Rheza Gandi Bawono**: Investigator, Data Administration, Coordinating Examination in Neurophysiology. **Rusdy Ghazali Malueka**: Conceptualization, Methodology, Supervision, Data Analysis, Writing – original draft.

## Declaration of Competing Interest

The authors declare that they have no known competing financial interests or personal relationships that could have appeared to influence the work reported in this paper.

## Figures and Tables

**Table:** 

No	VARIABEL/KODE	JAWABAN	No. Var
1	Nomor Penelitian		1
2	Nama		2
3	Umur (tahun)	…tahun	3
4	Alamat		4
5	Pendidikan terakhir		5
6	Tanggal pemeriksaan	…/…/…	6
7	Jenis kelamin:		7
	Laki-Laki Perempuan	(1) (2)	
8	Status perkawinan		8
	Kawin Tidak kawin	(1) (2)	
9	Pekerjaan		9
	- Lama kerja (tahun) - Jumlah jam kerja per hari - Pekerjaan sampingan	…tahun …jam	
10	Penggunaan alat pelindung diri		10
	- Selama bekerja - Kadang-kadang - Tidak memakai	(1) (2) (3)	
11	Tinggi badan (cm)	…	11
	Berat badan (kg)	…	
	Index massa tubuh	…	

## Data Availability

The data that has been used is confidential.

## Appendix

Kuesioner Hubungan Kadar Kholinesterase Darah dengan Kelainan Neurologi Petani Terpajan Pestisida di Kecamatan Ngablak Kabupaten Magelang Jawa Tengah.

**PENGETAHUAN TENTANG PESTISIDA**.

*Responden menjawab sebanyak-banyaknya jawaban yang dianggap benar dengan memberi tanda* (*√*).

1. Apa yang saudara ketahui pestisida?
  a. Zat untuk membunuh hama
  b. Zat untuk mengendalikan berbagai hama
  c. Zat untuk mencegah berbagai hama
  d. Zat untuk memberantas berbagai hama
  e. Zat untuk memberantas/mencegah binatang-binatang dan jasad renik dalam rumah tangga
2. a. Bentuk kering
  b. Bentuk umpan
  c. Bentuk butiran
  d. Bentuk cair
  Sebutkan macam bentuk formulasi pestisida menurut saudara ?
3. a. Aturan pakai atau dosis
  b. Petunjuk pertolongan bila terjadi keracunan
  c. Gejala-gejala keracunan
  d. Zat penawar racun (antidote)
  e. Nomor ijin produksi
  f. Tanggal kadaluwarsa
  Keterangan apa saja yang terdapat pada label Pestisida?
4. a. Pelindung kepala (helm/topi/caping)
  b. Pelindung pernafasan (masker)Pelindung badan (apron/overall)
  c. Pelindung tangan (glove)
  d. Pelindung kaki (sepatu boot)
  e. Pelindung mata (google)
  Pakaian apa saja yang seharusnya digunakan pada saat melakukan penyemprotan?
5. a. 1. Mulut
    2. Pernafasan
    3. Kulit
    Tahu, melalui:
  b. Tidak tahu
  Apakah saudara tahu bagaimana cara pestisida masuk ke dalam tubuh?
6. a. Mungkin
  b. Tidak mungkin
  Mungkinkah orang yang tidak pernah melakukan penyemprotan dapat mengalami keracunan pestisida?
7. a. Mulut
  b. Kulit
  c. Hidung
  d. Mata
  e. Luka
  Menurut saudara pestisida masuk ke dalam tubuh melalui apa saja ?
8. a. Pusing
  b. Muntah-muntah
  c. Kejang-kejang
  d. Ludah berlebihan
  e. Diare/mencret
  f. Mual-mual
  g. Keringat banyak
  h. Penglihatan kabur
  Sebutkan gejala dan tanda-tanda keracunan pestisida yang saudara ketahui ?
9. Menurut saudara bagaimana tindakan/cara pertolongan jika terjadi keracunan?
  a. Bila kena mata langsung dicuci dengan air bersih
  b. Bila terhisap langsung ke udara segar / luar rangan
  c. Meminum susu cair yang banyak
  d. Dibawa ke sarana kesehatan / Puskesmas
  e. Mencuci kulit yang terkena
  f. Pakaian yang terkena racun segera dilepaskan.
10. a. Kaleng/plastik dilubangi dahulu dan ditanam dalam tanah jauh dari sumber air
  b. Kertas bekas dibakar jauh dari permukiman
  Bagaimana cara membuang bekas kemasan / kaleng pestisida ?
11. a. Pestisida disimpan masih dalam kemasan aslinya
  b. Diletakkan dalam ruangan khusus yang ada ventilasinya
  c. Terhindar langsung dari sinar matahari
  d. Tidak disatukan dengan gudang makanan
  e. Ruangan penyimpanan harus terkunci
  f. Terpisah dengan dapur.
  Bagaimana cara penyimpanan pestisida ?
12. a. Dicuci dengan sabun
  b. Dicuci dengan air mengalir
  c. Tidak mencemari air bersih
  d. Tidak mencemari sungai
  Bagaimana cara saudara membersihkan alat-alat yang digunakan sesudah penyemprotan

Kuesioner Hubungan Kadar Kholinesterase Darah dengan Kelainan Neurologi Petani Terpajan Pestisida di Kecamatan Ngablak Kabupaten Magelang Jawa Tengah.

**KEPATUHAN PENGGUNAAN ALAT –ALAT PELINDUNG DAN HIGIENITAS**.

1. Apakah selama anda menyemprot selalu memakai kacamata sampai selesai?
  a. Ya
  b. Tidak
2. Apakah selama anda menyemprot selalu menggunakan sarung tangan sampai selesai?
  a. Ya
  b. Tidak
3. Apakah selama anda menyemprot selalu menggunakan celana panjang sampai selesai?
  a. Ya
  b. Tidak
4. Apakah selama anda menyemprot selalu menggunakan baju lengan panjang sampai selesai?
  a. Ya
  b. Tidak
5. Apakah selama anda menyemprot selalu menggunakan masker sampai selesai?
  a. Ya
  b. Tidak
6. Apakah selama anda menyemprot selalu menggunakan apron sampai selesai?
  a. Ya
  b. Tidak
7. Apakah selama anda menyemprot selalu menggunakan sepatu boot sampai selesai?
  a. Ya
  b. Tidak
8. Apakah selama anda menyemprot selalu menggunakan topi sampai selesai?
  a. Ya
  b. Tidak

## References

[1] Abang A.F., Kouamé C.M., Abang M., Hanna R., Fotso A.K. (2014). Assessing vegetable farmer knowledge of diseases and insect pests of vegetable and management practices under tropical conditions. Int. J. Veg. Sci..

[2] Leso V., Capitanelli I., Lops E.A., Ricciardi W., Iavicoli I. (2017). Occupational chemical exposure and diabetes mellitus risk. Toxicol. Ind. Health.

[3] Yadav S.S., Singh M.K., Yadav R.S. (2016). Organophosphates induced Alzheimer’s disease: an epigenetic aspect. J. Clin. Epigenet..

[4] Lerro C.C., Koutros S., Andreotti G., Friesen M.C., Alavanja M.C., Blair A. (2015). Organophosphate insecticide use and cancer incidence among spouses of pesticide applicators in the Agricultural Health Study. Occup. Environ. Med..

[5] Muñoz-Quezada M.T., Lucero B.A., Iglesias V.P., Muñoz M.P., Cornejo C.A., Achu E. (2016). Chronic exposure to organophosphate (OP) pesticides and neuropsychological functioning in farm workers: a review. Int. J. Occup. Environ. Health.

[6] Wesseling C., Keifer M., Ahlbom A., McConnell R., Moon J.-D., Rosenstock L. (2002). Long-term neurobehavioral effects of mild poisonings with organophosphate and n-methyl carbamate pesticides among banana workers. Int. J. Occup. Environ. Health.

[7] Prijanto T.B., Nurjazuli S., Sulistiyani S. (2009).

[8] Yuantari M.G.C., Setiani O., Nurjazuli N. (2009). Studi ekonomi lingkungan penggunaan pestisida dan dampaknya pada kesehatan petani di area pertanian hortikultura Desa Sumber Rejo Kecamatan Ngablak Kabupaten Magelang Jawa Tengah. J. Kesehat. Lingkung. Indones..

[9] Albers J.W., Garabrant D.H., Schweitzer S.J., Garrison R.P., Richardson R.J., Berent S. (2004). The effects of occupational exposure to chlorpyrifos on the peripheral nervous system: a prospective cohort study. Occup. Environ. Med..

[10] Karami-Mohajeri S., Nikfar S., Abdollahi M. (2014). A systematic review on the nerve–muscle electrophysiology in human organophosphorus pesticide exposure. Hum. Exp. Toxicol..

[11] World Health Organization, Sound Management of Pesticides and Diagnosis and Treatment of Pesticide Poisoning, Geneva, 2006.

[12] Ellman G.L., Courtney K.D., Andres V.J., Feather-Stone R.M. (1961). A new and rapid colorimetric determination of acetylcholinesterase activity. Biochem. Pharmacol..

[13] Zlatković M., Krstić N., Subota V., Bošković B., Vučinić S. (2017). Determination of reference values of acetyl and butyryl cholinesterase activities in Serbian healthy population. Vojnosanit. Pregl..

[14] Liveson J.A., Ma D.M. (1992).

[15] Barcenilla A., March L.M., Chen J.S., Sambrook P.N. (2012). Carpal tunnel syndrome and its relationship to occupation: a meta-analysis. Rheumatology.

[16] Darvesh S., Darvesh K.V., McDonald R.S., Mataija D., Walsh R., Mothana S., Lockridge O., Martin E. (2008). Carbamates with differential mechanism of inhibition toward acetylcholinesterase and butyrylcholinesterase. J. Med. Chem..

[17] Soderlund D.M. (2012). Molecular mechanisms of pyrethroid insecticide neurotoxicity: recent advances. Arch. Toxicol..

[18] Pathak M.K., Fareed M., Bihari V., Reddy M.M.K., Patel D.K., Mathur N. (2011). Nerve conduction studies in sprayers occupationally exposed to mixture of pesticides in a mango plantation at Lucknow, North India. Toxicol. Environ. Chem..

[19] Glynn P. (2006). A mechanism for organophosphate-induced delayed neuropathy. Toxicol. Lett..

[20] Boostani R., Mellat A., Afshari R., Derakhshan S., Saeedi M., Rafeemanesh E. (2014). Delayed polyneuropathy in farm sprayers due to chronic low dose pesticide exposure. Iran. Red Crescent Med. J..

[21] Zhang C., Sun Y., Hu R., Huang J., Huang X., Li Y. (2018). A comparison of the effects of agricultural pesticide uses on peripheral nerve conduction in China. Sci. Rep..

[22] Bernardes M.F., Pazin M., Pereira L.C., Dorta D.J. (2015). Impact of pesticides on environmental and human health. Toxicol. Stud. Cells Drugs Environ..

[23] Duirk S.E., Collette T.W. (2006). Degradation of chlorpyrifos in aqueous chlorine solutions: pathways, kinetics, and modeling. Environ. Sci. Technol..

[24] Lotti M., Moretto A. (2005). Organophosphate-induced delayed polyneuropathy. Toxicol. Rev..

[25] Wang Y., Li W., Martínez-Moral M.P., Sun H., Kannan K. (2019). Metabolites of organophosphate esters in urine from the United States: concentrations, temporal variability, and exposure assessment. Environ. Int..

[26] Elango K., Arunkumar P., Vijayalakshmi G. (2022). Factors affecting the toxicity of pesticides: an overview. Adv. Agric. Hortic. Sci..

